# A Low-Cost HPV Immunochromatographic Assay to Detect High-Grade Cervical Intraepithelial Neoplasia

**DOI:** 10.1371/journal.pone.0164892

**Published:** 2016-10-20

**Authors:** Vânia Sammartino Mariano, Adriana Tarlá Lorenzi, Cristovam Scapulatempo-Neto, Maíra Degiovani Stein, Julio Cesar Possati Resende, Márcio Antoniazzi, Luisa Lina Villa, José Eduardo Levi, Adhemar Longatto-Filho, José Humberto Tavares Guerreiro Fregnani

**Affiliations:** 1 Molecular Oncology Research Center, Barretos Cancer Hospital/Pio XII Foundation, Barretos, Sao Paulo, Brazil; 2 Pathology Department, Barretos Cancer Hospital/Pio XII Foundation, Barretos, Sao Paulo, Brazil; 3 Cancer Prevention Department, Barretos Cancer Hospital/Pio XII Foundation, Barretos, Sao Paulo, Brazil; 4 Department of Radiology and Oncology, Faculty of Medicine, University of Sao Paulo, Sao Paulo, Brazil; 5 Virology Laboratory, LIM 52.The Institute of Tropical Medicine of the University of Sao Paulo, Sao Paulo, Brazil; 6 Laboratory of Medical Research Laboratory, LIM 14.Department of Pathology, Faculty of Medicine, University of Sao Paulo, Sao Paulo, Brazil; 7 Life and Health Sciences Research Institute (ICVS), School of Health Sciences, University of Minho, Braga, Portugal; 8 ICVS/3B’s–PT Government Associate Laboratory, Braga/Guimaraes, Portugal; Bharathidasan University, INDIA

## Abstract

**Objective:**

To evaluate the reproducibility and accuracy of the HPV16/18-E6 test.

**Methods:**

The study population was comprised of 448 women with a previously abnormal Pap who were referred to the Barretos Cancer Hospital (Brazil) for diagnosis and treatment. Two cervical samples were collected immediately before colposcopy, one for the hr-HPV-DNA test and cytology and the other for the HPV16/18-E6 test using high-affinity monoclonal antibodies (mAb). Women with a histologic diagnosis of cervical intraepithelial neoplasia grade 2 or 3 were considered to be positive cases. Different strategies using a combination of screening methods (HPV-DNA) and triage tests (cytology and HPV16/18-E6) were also examined and compared.

**Results:**

The HPV16/18-E6 test exhibited a lower positivity rate compared with the HPV-DNA test (19.0% vs. 29.3%, p<0.001) and a moderate/high agreement (kappa = 0.68, 95%CI: 0.60–0.75). It also exhibited a significantly lower sensitivity for CIN2+ and CIN3+ detection compared to the HPV-DNA test and a significantly higher specificity. The HPV16/18-E6 test was no different from cytology in terms of sensitivity, but it exhibited a significantly higher specificity in comparison to ASCH+. A triage test after HPV-DNA detection using the HPV16/18-E6 test exhibited a significantly higher specificity compared with a triage test of ASCH+ to CIN2+ (91.8% vs. 87.4%, p = 0.04) and CIN3+ (88.6% vs. 84.0%, p = 0.05).

**Conclusion:**

The HPV16/18-E6 test exhibited moderate/high agreement with the HPV-DNA test but lower sensitivity and higher specificity for the detection of CIN2+ and CIN3+. In addition, its performance was quite similar to cytology, but because of the structural design addressed for the detection of HPV16/18-E6 protein, the test can miss some CIN2/3+ lesions caused by other high-risk HPV types.

## Introduction

Cervical cancer is the fourth most common cancer in females worldwide, with approximately 530,000 new cases and 275,000 deaths occurring annually. Approximately 85% of these cases occur in developing countries where organized screening programs and treatment modalities are not commonly available [1]. Cervical cancer is the third most common neoplasia in Brazilian women, affecting approximately 16,000 women annually [2].

The most important risk factor for cervical carcinogenesis is persistent infection by high-risk types of human papillomaviruses (hr-HPV) [3]. HPV infection is a very common sexually transmitted disease, and approximately 200 HPV types have been identified and categorized according to their oncogenic potential [4].

The implementation of cervical cytology-based screening strategies (Pap test) have reduced the incidence and mortality of cervical cancer in developed countries, but these outcomes have not changed significantly in developing countries, likely because of unsatisfactory adherence to screening programs, lack of cytology quality control and barriers to access to public health systems [5–8]. Replacement of the Pap test with hr-HPV testing is currently under consideration because of its high sensitivity and negative predictive value [9]. The U.S. Food and Drug Administration (FDA) recently approved hr-HPV testing as the primary screening tool for cervical cancer as a standalone test or associated with the Pap test, and World Health Organization (WHO) guidelines currently recommend hr-HPV testing in countries that do not have effective cytology testing-based screening programs [10]. HPV tests are generally user-friendly platforms that exhibit a high degree of reproducibility, and several tests with promising performance are currently available commercially [11–15]. However, HPV testing alone is burdened with a high number of false-positives due to its high sensitivity and low specificity rates. Nevertheless, most infections disappear within one year with no clinical consequences [16]. This high false- positive rate may be problematic for public health systems and the success of the implementation of screening programs in low- and middle-income settings [17,18]. Currently, most of these HPV tests are DNA-based assays, which are more useful for high workload scenarios because of the stability of DNA in cervical samples. However, screening strategies also detect cell changes and/or the presence of hr-HPV that will not necessarily result in cervical disease [19].

A low-cost immunochromatographic assay called the OncoE6 Cervical Test (Arbor Vita Corporation, Fremont, CA, USA) was developed to detect elevated expression of the hr-HPV 16/18E6 oncoproteins (HPV16/18-E6), which are necessary and sufficient for oncogenic transformation of cervical epithelial cells [20,21]. HPV types 16 and 18 are the most prevalent types in high-grade lesions of the uterine cervix, and these types cause approximately 70% of cervical cancers [22]. Although not yet commercially available nor approved by the FDA, preliminary results from other studies developed mainly in China are promising and demonstrate the E6 test as adjunctive treatment for patients; new data are expected soon [23]. The current study evaluated the reproducibility and accuracy of the HPV16/18-E6 assay.

## Materials and Methods

This prospective study enrolled women who were referred to the Prevention Department of Barretos Cancer Hospital (HCB), Barretos, SP, Brazil, and enrolled women aged 18 years or over who had recently undergone a Pap test that showed “atypical squamous cells—cannot excluded high-grade squamous intraepithelial lesion” or worse (ASCH+). Pregnant women were not eligible for the study.

This study was approved by the Research Ethics Committee of Barretos Cancer Hospital. All participants provided written informed consent, and all of the identifying information for the patients was maintained encrypted in a database to ensure data confidentiality and privacy of the participants. Arbor Vita Corporation donated all the OncoE6 kits. The senior author (JHF) performed all the analyses and confirmed that the company did not interfere in the analyses or interpretation of the data or manuscript writing.

Two cervical samples were collected using a Rovers brush (Rovers Medical Devices, Oss, The Netherlands) immediately before colposcopy. The first sample was preserved in a BD Surepath vial (Becton Dickinson, Burlington, NC, USA) for the hr-HPV-DNA test and cytology, and the other was placed in a ThinPrep vial (Hologic, Marlborough, MA, USA) for the HPV16/18-E6 assay. The former sample was stored at 4°C, and the latter was stored at -20°C. Subsequently, those women with cervical lesions observed upon colposcopy underwent biopsy and/or endocervical curettage (EC) according to the classification of the transformation zone. All the women with an unsatisfactory colposcopy underwent EC. For patients without colposcopic alterations, the exam was considered normal, no biopsy was performed and the results were considered negative. Two experienced gynecologists performed all the clinical examinations, and guided biopsies were performed based upon the colposcopic findings. The cytology slides were evaluated using a BD Focal Point system (Becton Dickinson, Burlington, NC, USA), and a senior cytotechnologist and cytopathologist analyzed any abnormal findings. A pathologist (CS-N) experienced in lower genital tract diseases blindly analyzed the tissue samples obtained by biopsy and/or EC. Cytology, and the results were classified based upon the criteria established in 2001 by the Bethesda System [24]. Histological diagnoses corresponded to the results of colposcopy-guided biopsies or cone specimens (whichever sample was worse); the histological alterations represented the gold standard for calculating the accuracy of the methods. To perform classification in two groups of high-grade lesions, we grouped the cases into CIN2+ (including CIN2, CIN3/carcinoma *in situ*, invasive carcinoma and carcinoma not otherwise specified) and CIN3+ (including CIN3/carcinoma *in situ*, invasive carcinoma and carcinoma not otherwise specified).

Residual samples from BD SurePath vials were used for the Cobas HPV test (Roche, Indianapolis, IN, USA), which is a qualitative multiplex assay that identifies HPV types 16 and 18 individually in addition to a pool of 12 other hr-HPVs(31, 33, 35, 39, 45, 51, 52, 56, 58, 59, 66 and 68).

**Fig 1:**
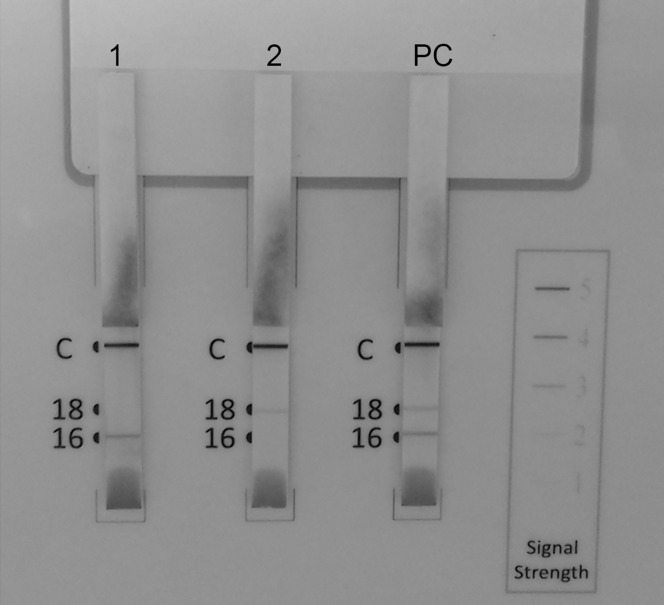
Examples of specimens using the HPV16/18-E6 test. (C) Control line; (16) HPV16-E6 line; (18) HPV-18-E6 line. Strip on left side (1): cervical sample HPV16 positive; Strip in the middle (2): cervical sample HPV18 positive; Strip on the right side (PC): positive control (HPV16/18 positive).

A ThinPrep sample was used to perform the OncoE6 Cervical Test (Arbor Vita Corporation, Fremont, CA, USA), which is an immunochromatographic assay that detects HPV16/18-E6 using a lateral flow format with high-affinity monoclonal antibodies (mAb) to oncoprotein from cell lysates generated from cervical swab specimens or methanol-based preservative solutions. Three test strips allowing for analysis of one individual clinical specimen and several units (of 3 test strips each) can be used in parallel by one operator. A control line is included on each strip, which allows for verification of detector reagent activity and proper sample solution migration up the test strip. Briefly, the cervical sample lysate is denoted by the appearance of a purple line at the specific location for HPV type on the strip, indicating a positive test (Fig 1). A trained technician conducted all the tests, and the detailed protocol is described elsewhere [23].

Sample size calculation was based on the following parameters: expected HPV16/18-E6 performance (sensitivity: 50%, specificity: 95%), prevalence of CIN2+ in the study population (25%, according to not published preliminary data of our service), precision (10%) and dropout rate (5%). According to these parameters, 429 women should be included in the study.

The McNemar test was used to compare the positivity rates of the HPV16/18-E6 test, HPV-DNA test and cytology. The kappa coefficient was calculated to evaluate the agreement between the tests. Sensitivity and specificity were based on the cervical biopsies and colposcopies, which were compared using the McNemar test. The area under the ROC curve (AUC) was also estimated, and the comparison between areas was performed using a nonparametric approach, as described by DeLong et al. [25]. Women with a histologically proven (colposcopy-guided biopsy or cone specimen, whichever was worse) diagnosis of CIN2+ or CIN3+were considered to be positive cases by the gold standard definition. Women with a histologically proven diagnosis of low-grade intraepithelial neoplasia, cervicitis or metaplasia were considered negative (< CIN2). Women with no significant colposcopic findings did not undergo cervical biopsy, and they were considered to be negative. Different strategies using a combination of screening methods (HPV-DNA) and triage tests (cytology and HPV16/18-E6) were examined and compared. The significance level was set at 5% for all the tests. All the analyses were performed using SPSS version 20 and MedCalc version 13.

## Results

The study included 448 women aged 18 to 89 years (median age = 39.3 years). Table 1 shows the results of the cytology (collected immediately before colposcopy), histology diagnosis and HPV test results (DNA and E6 protein). Cytology was positive in 262/446 (58.7%) women, and a histological diagnosis of CIN2 and CIN3/carcinoma *in situ* was obtained in 47/368 (12.8%) and 59/368 (16.0%) cases, respectively. Invasive carcinoma/not otherwise specified was reported in 13/368 women (3.5%).

**Table 1:** Number and percentage of cases according to cervical cytology, histology diagnosis, HPV-DNA test and HPV16/18-E6 test.

	N	(%)
Cervical cytology (*1)		
Negative	183	(41.0)
Atypical squamous cells of undetermined significance (ASCUS)	64	(14.3)
Atypical squamous cells of undetermined significance (ASCH)	50	(11.2)
Atypical glandular cells (AGC)	13	(2.9)
Low-grade squamous intraepithelial lesions (LSIL)	73	(16.4)
High-grade squamous intraepithelial lesions (HSIL)	62	(13.9)
Unsatisfactory	1	(0.2)
Histology diagnosis (*2)		
Cervicitis or metaplasia	148	(40.2)
CIN1	101	(27.5)
CIN2	47	(12.8)
CIN3 / Carcinoma in situ	59	(16.0)
Invasive carcinoma / Not otherwise specified (NOS)	13	(3.5)
HPV-DNA test (*3)		
Negative	182	(40.7)
Positive	265	(59.3)
HPV16/18-E6 test (*4)		
Negative	362	(80.8)
Positive	86	(19.2)

(*1) Cytology collected immediately before colposcopy. Cytology was not performed in two cases.

(*2) Diagnosis obtained from colposcopy-guided biopsy or cone specimen, whichever was worse. Biopsy was not performed in 80 cases because there was nothing found on colposcopy.

(*3) One case had no residual sample to test. HPV16 was detected in 109 cases, HPV18 in 27, and other high-risk types in 183. Co-infection was identified in 50 cases.

(*4) HPV16-E6 was detected in 70 cases and HPV18-E6 in 16. No HPV16/HPV18 co-infection was detected using the OncoE6 test.

HPV-DNA tests were positive in 265/448 (59.2%) women. Among cases with positive results, HPV16-DNA was detected in 109 (41.1%), HPV18-DNA in 27 (10.2%), and other hr-types were detected in 183 (69.1%). Co-infection (when more than one type of hr-HPV was detected in the same sample) was observed in 50/448 (11.2%) cases analyzed using Cobas. The HPV16/18-E6 test exhibited a positivity rate of 19.2% (86/448). HPV16-E6 was detected in 70 positive cases (81.4%), and HPV18-E6 was detected in 16 positive cases (18.6%). The test did not identify HPV16/HPV18 co-infection.

Table 2 shows comparisons of the HPV16/18-E6 test to HPV-DNA. The HPV16/18-E6 test exhibited a lower positivity rate compared with the HPV-DNA test (19.0% vs. 29.3%, McNemar test: p<0.001). Considering the cases with CIN2+ and CIN3+ diagnoses, the positivity rates were 49.2% vs. 63.6% and 59.2 vs. 73.2% for the HPV16/18-E6 and HPV-DNA tests (McNemar tests: p<0.001), respectively. Kappa indexes were moderate/high in all the analyses (whole study population: 0.68, 95%CI: 0.60–0.75; population with CIN2+: 0.68, 95%CI: 0.55–0.81; population with CIN3+: 0.69, 95%CI: 0.52–0.86).

**Table 2:** Agreement and association analyses of HPV16/18-E6 and HPV-DNA tests. CI, confidence interval.

Analysis		HPV16/18-E6 test			Kappa index(95% CI)	McNemar test (p value)
		HPV 16/18 (-)	HPV 16/18 (+)			
**All cases**	**HPV-DNA test**			Total		
	HPV 16/18(-)	312	4	316 (70.7%)	0.68 (0.60–0.75)	< 0.001
	HPV 16/18(+)	50	81	131 (29.3%)		
	Total	362 (81.0%)	85 (19.0%)	447 (100.0%)		
**CIN2+**	**HPV-DNA test**			Total		
	HPV 16/18(-)	42	1	43 (36.4%)	0.68 (0.55–0.81)	< 0.001
	HPV 16/18(+)	18	57	75 (63.6%)		
	Total	60 (50.8%)	58 (49.2%)	118 (100.0%)		
**CIN3+**	**HPV-DNA test**			Total		
	HPV 16/18(-)	19	0	19 (26.8%)	0.69 (0.52–0.86)	< 0.001
	HPV 16/18(+)	10	42	52 (73.2%)		
	Total	29 (40.8%)	42 (59.2%)	71 (100.0%)		

Tables 3 and 4 show the performance of the HPV16/18-E6 test, HPV-DNA test and cytology for the detection of CIN2+and CIN3+, respectively. The HPV16/18-E6 test exhibited a significantly lower sensitivity for CIN2+ detection compared with the HPV-DNA (any type of hr-HPV and HPV16/18) but a significantly higher specificity. The HPV16/18-E6 test showed no difference from cytology in sensitivity but exhibited a significantly higher specificity in comparison to ASCH+. HSIL exhibited slightly better specificity compared with HPV16/18-E6, but this difference was not significant (p = 0.07). The performance of HPV16/18-E6 exhibited a similar profile for CIN3+ diagnosis as for CIN2+.

**Table 3:** Performance of HPV16/18-E6 test, HPV-DNA test and cytology for the detection of CIN2+. CI, confidence interval; AUC, area under ROC curve; ASCH+, Atypical Squamous Cells of Undetermined Significance or worse; HSIL, High-grade Squamous Intraepithelial Lesion. CIN2+: CIN2, CIN3, carcinoma in situ, invasive carcinoma, carcinoma not otherwise specified.

Criteria	Sensitivity	Specificity	AUC
	% (95% CI)	% (95% CI)	(95% CI)
HPV16/18-E6 test	**49.6 (40.3–58.9)**	**91.8 (88.3–94.5)**	**0.71 (0.65–0.77)**
HPV-DNA (any high-risk)	94.9 (89.3–98.1) *	53.5 (47.9–59.0) *	0.74 (0.70–0.79)
HPV-DNA 16/18	63.3 (53.8–72.0) *	83.0 (78.5–86.9) *	0.73 (0.68–0.79)
HPV-DNA non-16/18	51.7 (42.3–61.0)	62.9 (57.5–68.2) *	0.57 (0.51–0.63) *
Pap test: ASCH +	59.0 (49.5–68.0)	82.9 (78.4–86.8) *	0.71 (0.65–0.77)
Pap test: HSIL	40.2 (31.2–49.6)	95.4 (92.6–97.4) **	0.68 (0.62–0.74)

(*) There is a statistically significant difference compared with the HPV16/18-E6 test.

(**) There is a marginal significance compared with the HPV16/18-E6 test (p = 0.07).

**Table 4:** Performance of HPV16/18-E6 test, HPV-DNA test and cytology for the detection of CIN3+. CI, confidence interval; AUC, area under ROC curve; ASCH+, Atypical Squamous Cells of Undetermined Significance or worse; HSIL, High-grade Squamous Intraepithelial Lesion. CIN3+: CIN3, carcinoma in situ, invasive carcinoma, carcinoma not otherwise specified.

Criteria	Sensitivity	Specificity	AUC
	% (95% CI)	% (95% CI)	(95% CI)
HPV16/18-E6 test	**59.7 (47.6–71.1)**	**88.6 (84.9–91.6)**	**0.74 (0.67–0.81)**
HPV-DNA (any high-risk)	98.6 (92.4–100) *	48.1 (43.0–53.3) *	0.73 (0.68–0.78)
HPV-DNA 16/18	72.9 (60.9–82.8) *	79.0 (74.5–83.0) *	0.76 (0.70–0.82)
HPV-DNA non-16/18	42.3 (30.6–54.6)	59.3 (54.2–64.3) *	0.51 (0.43–0.58) *
Pap test: ASCH +	70.0 (57.9–80.4)	79.7 (75.3–83.7) *	0.75 (0.68–0.82)
Pap test: HSIL	48.6 (36.4–60.8)	92.5 (89.4–95.0) **	0.71 (0.63–0.78)

(*) There is a statistically significant difference compared with the HPV16/18-E6 test.

(**) There is a marginal significance compared with the HPV16/18-E6 test (p = 0.07).

Tables 5 and 6 compare the effects of cytology or HPV16/18-E6 tests on the management of HPV DNA-positive women (triage) for CIN2+ and CIN3+, respectively. The addition of a triage test (cytology or HPV16/18-E6) decreased the sensitivity of the screening strategy but improved specificity. A triage test after HPV-DNA detection using theHPV16/18-E6 test exhibited a significantly higher specificity compared to a triage test of ASCH+ to CIN2+ (91.8% vs. 87.4%, McNemar test: p = 0.04) and CIN3+ (88.6% vs. 84.0%, McNemar test: p = 0.05). HPV16/18-E6 exhibited a significantly lower specificity compared to a triage test using HSIL (CIN2+: 91.8% vs. 95.7%, McNemar test: p = 0.04; CIN3+: 88.6% vs. 93.1, McNemar test: p = 0.04). However, we did not observe any statistically significant differences in sensitivity when the triage test was performed using HPV16/18-E6 or cytology (ASCH+ or HSIL).

**Table 5:** Performance of different strategies for the detection of CIN2+. CI, confidence interval; AUC, area under ROC curve; ASCH+, Atypical Squamous Cells of Undetermined Significance or worse; HSIL, High-grade Squamous Intraepithelial Lesion.

Screening	Triage	Sensitivity	Specificity	AUC
		% (95%CI)	% (95%CI)	(95%CI)
HPV-DNA test (any high-risk HPV)	HPV16/18-E6 test	49.2 (39.8–58.5)	91.8 (88.3–94.5)	0.71 (0.64–0.77)
HPV-DNA test (any high-risk HPV)	ASCH +	58.1 (48.6–67.2)	87.4 (83.5–90.9) *	0.73 (0.67–0.79)
HPV-DNA test (any high-risk HPV)	HSIL	39.4 (30.4–48.8)	95.7 (93.0–97.7) *	0.68 (0.61–0.74)
HPV-DNA test (any high-risk HPV)	None	94.9 (89.3–98.1) *	53.5 (47.9–59.0) *	0.74 (0.70–0.79)

(*) There is a statistically significant difference compared with the reference strategy, i.e., detection of HPV-DNA (any high-risk type) followed by detection of HPV-Oncoprotein E6 (p<0.05).

**Table 6:** Performance of different strategies for the detection of CIN3+. CI, confidence interval; AUC, area under ROC curve; ASCH+, Atypical Squamous Cells of Undetermined Significance or worse; HSIL, High-grade Squamous Intraepithelial Lesion.

Screening	Triage	Sensitivity	Specificity	AUC
		% (95%CI)	% (95%CI)	(95%CI)
HPV-DNA (any type)	HPV-E6 test	59.2 (46.8–70.7)	88.6 (84.9–91.6)	0.74 (0.67–0.81)
HPV-DNA (any type)	ASCH +	70,0 (57.9–80.4)	84.0 (79.9–87.6) *	0.77 (0.70–0.84)
HPV-DNA (any type)	HSIL +	48.6 (36.4–60.8)	93.1 (90.0–95.4) *	0.71 (0.63–0.79)
HPV-DNA (any type)	None	98.6 (92.4–100) *	48.1 (43.0–53.3) *	0.73 (0.68–0.78)

(*) There is a statistically significant difference compared with the reference strategy, i.e., detection of HPV-DNA (any high-risk type) followed by detection of HPV-Oncoprotein E6 (p<0.05).

## Discussion

The HPV test is a useful approach to cervical cancer screening based on the knowledge that hr-HPV chronic infection is the key risk factor for the disease [26–28]. Negative HPV test is informative since HPV causes virtually all cervical cancers. However, a positive test does not necessarily represent disease because only a fraction of all HPV-positive women will develop high-grade disease. Therefore, the detection of HPV-DNA in cytological samples does not indicate a mandatory diagnosis of CIN or cancer, but elevated expression of HPV16/18-E6 is considered a necessary step for oncogenic transformation of cervical epithelial cells [29]. Accordingly, the detection of HPV-DNA associated with high levels of HPV16/18-E6 may be an interesting approach for CIN detection in cervical cancer prevention strategies.

We selected women with previously abnormal Pap smears, i.e., a high-risk population for cervical abnormality. The prevalence rate of HPV infection as detected using the HPV-DNA assay was impressively high (59.3%) because of this selection bias. These results differ from the prevalence of HPV reported by Lorenzi et al. (12%) in a low-risk population in Brazil [30]. However, this bias did not affect the conclusions of the study because disease prevalence is not expected to modify the sensitivity or specificity. High agreement was observed between HPV16/18-E6 detection and the presence of HPV-DNA in the cytology samples. However, the positivity rate of HPV infection was higher in the DNA assay compared with the immunochromatographic method. This result is not surprising because molecular tests are more sensitive than protein-based assays.

The current study showed that the detection of HPV16/18-E6 in cytological samples was highly specific but exhibited a low sensitivity for the detection of CIN2+ or CIN3+. Also, there was no significant difference in the sensitivity to ASCH+ or HSIL compared to cytology, but the specificity was significantly higher for the HPV16/18-E6 assay compared to ASCH+. The sensitivity of the HPV-DNA test to detect CIN2+ or CIN3+ was higher than that of the HPV16/18-E6 assay, but the specificity was better in the latter method. Only HSIL exhibited a slightly higher specificity compared with HPV16/18-E6 detection. Although the HPV16/18-E6 tests had exhibited similar performance to cervical cytology, the HPV16/18-E6 test is a point-of-care solution, which may be an alternative tool for use in low-income settings with poor cytology screening programs. Moreover, the HPV16/18-E6 assay does not require complex equipment and results can be obtained approximately 2.5 hours after sample collection. Although the HPV16/18-E6 has just been commercially available, the price of the test will probably be higher than that for cytology, which could be a barrier to implementation in low-middle income countries. Cost-effectiveness analyses are mandatory in further studies.

Notably, this study demonstrated that HPV16/18-E6 detection exhibited the same sensitivity for ASCH+ as a triage method to detect CIN2+ and CIN3+ after a positive HPV-DNA test but with better specificity.

Some of the CIN2+ that was not detected by the HPV16/18-E6 test may not progress to cancer, at least theoretically. McCredie [31] reported that most young women with early CIN3 (and CIN2) without treatment have a cumulative 30-year risk of invasive cancer of 31.3% (95% CI: 22.7–42.3), but other women with cytological evidence of persistent CIN3 within 2 years of diagnosis have a cumulative risk of 50.3% (95% CI: 37.3–64.9). Unfortunately, it is difficult to know what fraction of the early, small CIN3 lesions will persist and become large CIN3 lesions. Only a cohort study could answer this issue. However, one question remains: would it be ethically acceptable not to offer any treatment to women with a confirmed diagnoses of CIN3 and HPV16/18 infection?

Zhao et al. [32] analyzed the performance of the HPV16/18-E6 test (OncoE6™ Cervical Test, Arbor Vita, USA) and two HPV-DNA-based assays (CareHPV, Qiagen and Hybrid Capture, Qiagen) for the detection of CIN2+ and CIN3+ in a large cohort of more than 7,543 women living in rural China. Three samples were collected from these women. One sample was obtained by self-collection (vaginal material), and a clinician collected the other two samples (cervical material). HPV16/18-E6 was evaluated only in the clinician-collected samples, and the tests exhibited lower sensitivity rates for detection of CIN2+ (42.4%) and CIN3+ (53.5%) and higher specificity (CIN2+: 99.1%, CIN3+: 98.9%) than did our study. The differences in sensitivity and specificity compared to our study may be explained by the gold standard criteria used by Zhao et al. Approximately 10% of all women with a negative screen underwent a rigorous colposcopy, including a biopsy protocol. We did not biopsy all women, and positive cases may have been missed in women with a negative colposcopy, which may have biased the performance of the HPV16/18-E6 test. This verification bias certainly overestimated the sensitivity and underestimated the specificity [33]. However, the HPV16/18-E6 test exhibited higher specificity than ASCH+ and HPV-DNA detection in the current study, despite the verification bias.

Several studies have suggested that the detection of E6/E7 oncogene transcripts of high-risk HPV types would provide higher specificity in the screening of the risk for the development of high-grade lesions and squamous cell carcinomas [34–36]. Three messenger RNA (mRNA) tests for oncogene E6/E7 investigation are commercially available, but only NucliSENS Easy Q HPV™ (bioMérieux, Rome, Italy) has been approved by the FDA. This technique is based on nucleic acid sequence–based amplification, which utilizes beacon probes for real-time detection and typing of E6/E7 mRNA from HPV16, 18, 31, 33 and 45 [37]. This procedure exhibits higher specificity but lower sensitivity than DNA-based assays [34,37–40]. Cattani et al. [41] reported a lower sensitivity of mRNA assays using NucliSENS Easy Q™ compared with a DNA assay in high-grade lesions, but the specificity of the mRNA was higher. These results suggest that RNA assays may be used as a biomarker for the progression of neoplasia or a triage test after HPV-DNA detection [42]. However, no study compared HPV16/18-E6 and mRNA performance.

HPV16/18-E6 detection also exhibits some limitations. The test was not able to identify HPV16/18 co-infection, at least not in this study population. The authors did not find any plausible explanation for this. Moreover, the HPV16/18-E6 test is not performed in a high throughput fashion, and it only targets one type of oncoprotein of the two HPV types [43]. The E7 oncoprotein is also involved in cervical cell transformation [44], and other types of hr-HPV are also associated with carcinogenesis [45]. Oliveira et al. [46] defined the distribution of hr-HPV types in fresh samples from invasive cervical carcinoma in Brazil using a Linear Array approach. These authors noted the following HPV detection rates in the tumors: HPV16 (77.6%), HPV18 (12.3%), HPV31 (8.8%), HPV33 (7.1%) and HPV35 (5.9%). Notably, HPV types 31, 33 and 35 were also detected as a single infection in 7.0%, 3.9% and 1.5%, respectively. Using polymerase chain reaction to detect HPV-DNA in 199 histologically confirmed incident invasive cervical cancer cases, 66% of the cases were positive for HPV types 16, 18, 31 and 33 together [47]. De Sanjose et al. [48] found HPV types 16 and 18 in 6,357 of 8,977 cases (71%) of invasive cervical cancers according to the distribution of HPV genotypes in invasive cervical cancer from 38 countries in Europe, North America, central South America, Africa, Asia, and Oceania. HPV types 16, 18, and 45 were detected in 443 of 470 cases (94%, 92–96) of cervical adenocarcinomas. The relationship between HPV genotypes and CIN or invasive cervical lesion was assessed using reverse hybridization, and HPV16 was the most prevalent type in 142 cases (49%), followed by HPV types 58, 52, 31, 35 and 33 [49]. These data suggest that subsequent versions of the HPV-E6 tests must strongly consider evaluation of the oncoproteins of hr-HPV types beyond 16 and 18.

In conclusion, the HPV16/18-E6 test exhibited moderate/high agreement with the HPV-DNA test but lower sensitivity and higher specificity for the detection of CIN2+ and CIN3+. In addition, its performance is quite similar to that of cytology, but because of the structural design addressed for the detection of the HPV16/18-E6 protein, the test can miss some CIN2/3+ lesions caused by other high-risk HPV types. Nevertheless, the HPV16/18-E6 test is a point-of-care solution that might be an alternative for cervical cancer prevention in low-income settings in which a cytology-based screening is poorly conducted or not implemented.
